# Structure determination of microbial metabolites by the crystalline sponge method†
†Electronic supplementary information (ESI) available: Details of sample preparation and crystallographic analysis. CCDC 1451761–1451763. For ESI and crystallographic data in CIF or other electronic format see DOI: 10.1039/c6sc00594b


**DOI:** 10.1039/c6sc00594b

**Published:** 2016-02-24

**Authors:** Yasuhide Inokuma, Tomoya Ukegawa, Manabu Hoshino, Makoto Fujita

**Affiliations:** a Department of Applied Chemistry , School of Engineering , The University of Tokyo , 7-3-1 Hongo, Bunkyo-ku , Tokyo 113-8656 , Japan . Email: mfujita@appchem.t.u-tokyo.ac.jp; b JST PRESTO , 4-1-8 Honcho , Kawaguchi , Saitama 332-0012 , Japan; c JST ACCEL , 4-1-8 Honcho , Kawaguchi , Saitama 332-0012 , Japan

## Abstract

The structures of metabolites produced in microgram quantities by enzymatic reductions with baker's yeast were analyzed using the crystalline sponge method. The X-ray data provided reliable structures for trace metabolites including their relative and absolute stereochemistries that are not fully addressed by conventional NMR and LC-MS analyses. Technically, combining two or more chromatographic purification techniques is essential because, unlike abundant synthetic compounds, extracted metabolites contain many low level UV-silent impurities. The crystalline sponge method coupled with HPLC purification (LC-SCD) would thus be a useful method for metabolic analysis and drug discovery.

## Abstract 2

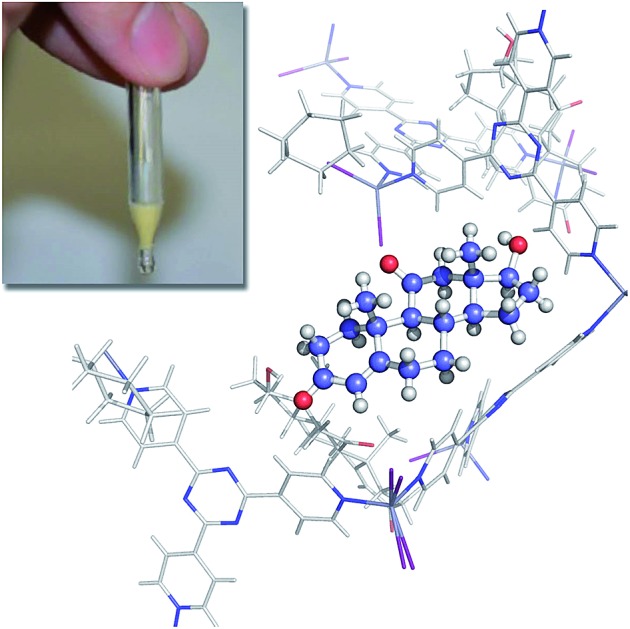
The structures of metabolites produced in microgram quantities by enzymatic reductions with baker's yeast were analyzed using the crystalline sponge method. The crystalline sponge method coupled with HPLC purification would be a useful method for metabolic analysis and drug discovery.

## Introduction

The structural information of metabolites is key to understanding enigmatic cellular processes and for drug design.^1^ Important insights into active enzymes, gene expression of RNA sequences, and biochemical reactions can be extracted when the structures of metabolites are fully characterized. They are in most cases produced in minute quantities as low level components of a complex mixture. Combinations of spectroscopic measurements (*e.g.* NMR and MS) and separation techniques (*e.g.* GC and LC) have been frequently used and established to elucidate the structures of scarce metabolites from a mixture. However, full characterization of their structures, including their absolute stereochemistry, is still a laborious task because of limited sample supply.

The crystalline sponge method^2^,^3^ is a recently developed technique to prepare single crystal samples for X-ray crystallographic analysis of trace and non-crystalline compounds. Given the low limit of the requisite sample amount (<0.1 μg), the method can be an innovative analytical tool for trace metabolites, but has only been used to examine abundant synthetic compounds in the past.^4^,^5^ The application of the method to bio-synthetically produced scarce metabolites is challenging because, unlike synthetic samples, extracted metabolites contain many unpredictable impurities. The major advantage of the crystalline sponge method is that a microgram level quantity of samples is sufficient to be analyzed. In this paper, we demonstrate that the microbial metabolites obtained only in microgram quantities are fully characterized using the crystalline sponge method coupled with HPLC separation (LC-SCD analysis).

## Results and discussion

As a model case of metabolic analysis using a crystalline sponge [(ZnI_2_)_3_(tpt)_2_·(*c*-C_6_H_12_)_*x*_]_*n*_ (**1**; tpt = tris(4-pyridyl)-1,3,5-triazine),3a,^6^ we conducted reductive dechlorination of 1,1-bis(4-chlorophenyl)-2,2,2-trichloroethane (DDT; **2**) by reductases found in baker's yeast. When compound **2** (100 mg) was treated with fermenting baker's yeast (dry yeast (11.2 g) and sucrose (23.5 g) in 100 ml of water) at 30 °C for 1 week, a small amount of a metabolic product (∼1.3% yield based on UV spectroscopy) was detected in the ether extract obtained from the mixture. LDI-TOF MS spectrometry indicated the loss of one chlorine atom (*m*/*z* = 320.040; calcd for C_14_H_10_Cl_4_^+^ [**2** – Cl + H]^+^: 319.9507) from **2**, suggesting the formation of 1,1-bis(4-chlorophenyl)-2,2-dichloroethane (DDD; **3**). The formation of **3** in a low yield is consistent with the previous report.^7^ However, another dechlorinated compound, **4**, cannot be excluded as the product based solely on MS information.

To unambiguously determine the structure of **3**, the crude extract was subjected to the LC-SCD analysis. After pre-purification of the ether extract (*ca.* 15 μg) using PTLC, the analytical sample was further purified using HPLC with a narrow collection time window (Fig. S3†). This purification protocol is particularly important, or else the HPLC-separated sample was considerably contaminated with many UV-silent impurities. In fact, no satisfactory results were obtained in our earlier attempts in the crystalline sponge analysis when the crude extract was directly purified using HPLC with a wide collection window (Fig. S5†).

The HPLC separation system was slightly modified so that fraction collection and subsequent guest soaking could be done in the same microvial (Fig. S1†). The fraction was directly received by a microvial and, after the evaporation of the eluent (hexane), one crystal of crystalline sponge **1** and a solvent were added therein and the vial was allowed to stand at room temperature for 3 d for guest soaking.

**Fig. 1:**
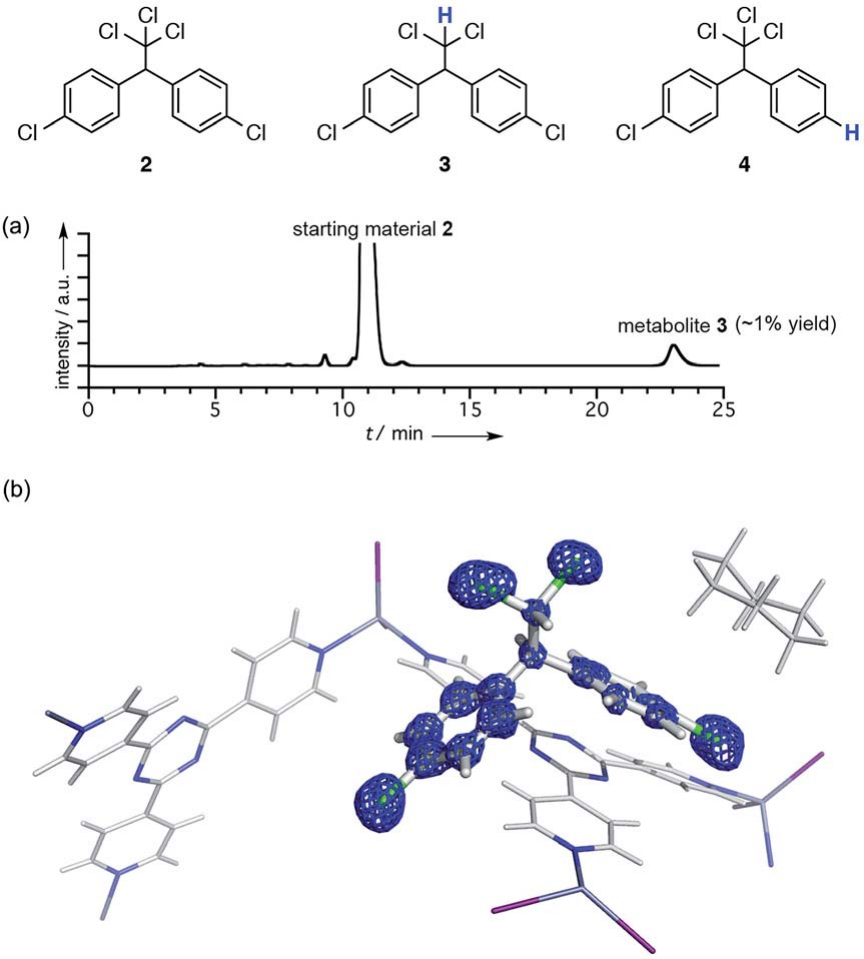
(a) HPLC chromatogram of the organic extract from the baker's yeast reaction. (b) The electron density map *F*_0_ (contoured at the 0.8*σ* level) around the binding site of metabolite **3** (guest A) in the pores of crystalline sponge **1**.

The crystallographic analysis confirmed the structure of the metabolite to be **3** (Fig. 1). The crystal structure revealed that there are two major binding sites for guest **3** in the pores of the crystalline sponge **1**.‡
‡Crystallographic data for **1·3**: C_36_H_24_N_12_Zn_3_I_6_·(C_14_H_10_Cl_4_)·(C_7_H_4_Cl_2_)·2.5(C_3_H_6_), *M* = 2230.02, colorless block, 0.23 × 0.11 × 0.09 mm^3^, monoclinic, space group *C*2/*c*, *a* = 35.7365(13) Å, *b* = 15.0610(5) Å, *c* = 30.9345(11) Å, *β* = 104.141(7)°, *V* = 16 145.2(11) Å^3^, *Z* = 8, *D*_c_ = 1.835 g cm^–3^, *T* = 93(2) K, 2.989° < *θ* < 27.468°, 17 652 unique reflections out of 88 524 with *I* > 2*σ*(*I*), GoF = 1.063, final *R* factors *R*_1_ = 0.0780, and w*R*_2_ = 0.2736 for all data, CCDC deposit number 1451761. One is located near a tpt ligand where guest **3** was observed with 100% occupancy (guest A; Fig. S5†). Another one is located on a crystallographic twofold axis where guest **3** was statistically disordered (guest B). A better resolution was obtained for A, and the electron density map *F*_0_ clearly shows the structure of **3** (Fig. 1b). Slightly high *R*_1_ and w*R*_2_ values (0.0780 and 0.2736, respectively) were obtained presumably due to very minor disorder in **3**, which cannot be modeled yet contributes to the residual electron density significantly because of the heavy atom (Cl).

When a prochiral functional group is enzymatically reduced to a chiral one, the stereochemical issues are of major concern for the structural characterization. Moreover, with the product(s) isolated only in microgram quantities, NMR analyses (particularly, ^13^C or 2D NMR) become difficult even for determination of the relative stereochemistry. The absolute stereochemistry can hardly be addressed by any spectroscopic methods unless empirical rules or data for authentic compounds are available. Thus, we applied the LC-SCD analysis for the full structural characterization (including absolute stereochemistry) of a metabolite from tetralone **5**. This compound exists as a racemate due to rapid enolization at C1 and can give four possible stereoisomers (including enantiomers) upon reduction of the carbonyl group at C2.

**Fig. 2:**
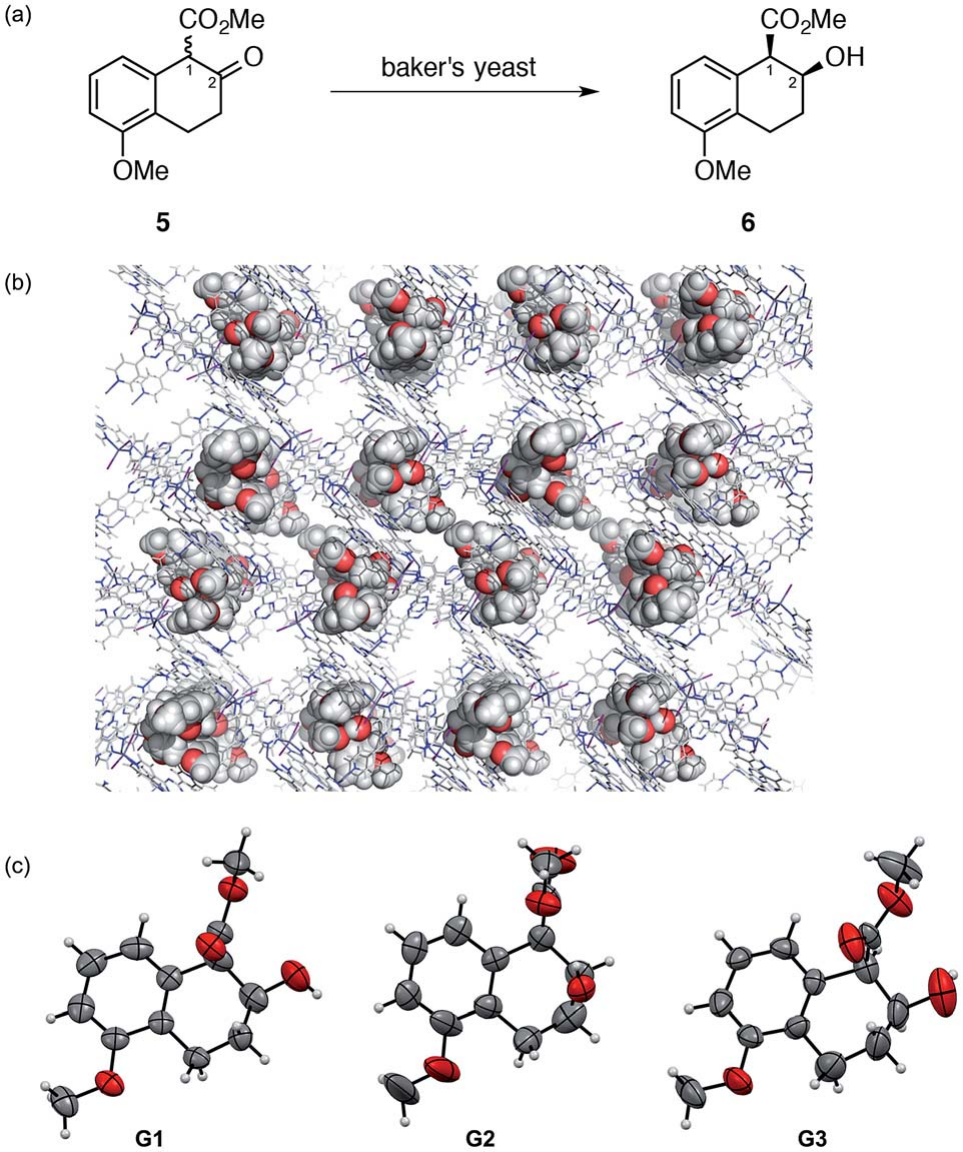
(a) Reduction of tetralone **5** by baker's yeast. (b) Crystal structure of metabolite **6**-incorporated crystalline sponge **1** viewing along the *b*-axis (metabolite **6**: CPK model, others: stick model, disordered guests/solvents were omitted for clarity). (c) ORTEP drawings of **G1–G3** at the 50% probability level.

Treating tetralone **5** (100 mg) with baker's yeast (11.2 g) for 18 h gave analytically pure chiral alcohol **6** in 10 μg quantity after isolation using HPLC. The relative stereochemistry of **6** was confirmed to be *cis* by comparison with both an authentic *cis*–*trans* mixture obtained by the NaBH_4_ reduction of **5** and literature reported spectroscopic data.^8^ Chiral HPLC analysis indicated >98% ee for the isolated *cis* isomer **6**. An inclusion crystal **1·6** was prepared by soaking a crystal of **1** in a cyclohexane/1,2-dichloroethane (v/v = 9 : 1) solution of **6**. The crystal structure was solved with a non-centrosymmetric space group *C*2.§
§Crystallographic data for **1·6**: C_72_H_48_I_12_N_24_Zn_6_·3.79(C_13_H_16_O_4_)·1.93(C_6_H_12_), *M* = 4220.24, colorless rod, 0.18 × 0.11 × 0.06 mm^3^, monoclinic, space group *C*2, *a* = 36.3783(8) Å, *b* = 14.6755(2) Å, *c* = 31.2278(6) Å, *β* = 103.184(2)°, *V* = 16 232.2(5) Å^3^, *Z* = 4, *D*_c_ = 1.727 g cm^–3^, *T* = 100(2) K, 4.1720° < *θ* < 74.0140°, 31 961 unique reflections out of 69 980 with *I* > 2*σ*(*I*), GoF = 1.031, final *R* factors *R*_1_ = 0.0511, and w*R*_2_ = 0.1396 for all data, Flack parameter (Parsons) = 0.010(4), CCDC deposit number 1451762. A reasonable Parsons' Flack parameter value of 0.010(4) was obtained and the final *R*_1_ and w*R*_2_ values were 0.0511 and 0.1396, respectively. In an asymmetric unit, three independent guest molecules **6** (**G1–G3** in Fig. 2) were clearly observed with ∼100% occupancy. All of the observed molecules **6** (**G1–G3**) show a 1*R*,2*S* absolute configuration, consistent with a previous report,^8^ in which an absolute configuration was speculated based on the absolute stereochemistry of analogous compounds.^8^ Our crystallographic study provided unquestionable proof for the absolute configuration of **6**. From this, a plausible reaction mechanism to give **6** is suggested to involve reduction from the *Re* face of a 1*R* isomer of **5** that is kinetically resolved within the enzyme pocket.

Conformational analysis of the observed guests **G1–G3** is worthy of additional discussion. The methoxycarbonyl groups in **G1** and **G3** are located at the pseudo-axial position, while that in **G2** is at the pseudo-equatorial position. For **G2**, intramolecular hydrogen bonding between the OH and COOMe groups was indicated by the short O_O–H_–O_C=O_ distance (∼2.8 Å) that may stabilize this conformation. Presumably, this conformer exists in solution and the crystalline sponge traps both conformers at different binding sites, enabling the concomitant observation of both conformers.

The structural and stereochemical analysis of a metabolite from adrenosterone (**7**) provided a more challenging task because three prochiral carbonyl carbons at C3, C11, and C17 can in principle generate 26 different compounds upon reduction of the carbonyl group(s). When steroid **7** was metabolized by baker's yeast on a 200 μg scale, HPLC analysis revealed the exclusive formation of a single metabolic product (hereafter denoted as **8**).

The parent ion peak of metabolite **8** was observed at *m*/*z* = 325.1781 (calcd for C_19_H_26_NaO_3_^+^: 325.1780). The increase in mass unit by 2.0 Da from **7** suggests a mono-hydrogenation of **7** at either a carbonyl or C=C group. The ^1^H NMR spectrum of **8** was, unfortunately, not conclusively able to determine its structure and stereochemistry because of overlapping signals. The unequivocal structure determination of **8** can be achieved, given that **8** is obtained only in microgram quantity, most reliably by the LC-SCD analysis.

**Fig. 3:**
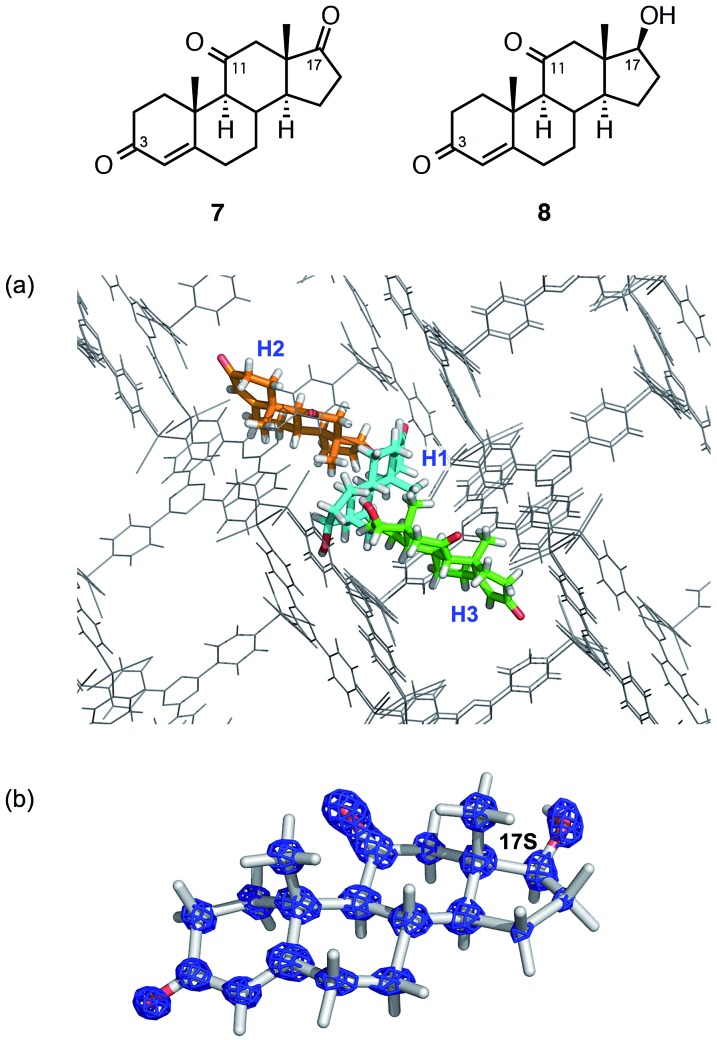
(a) Guest binding sites for metabolite **8** (**H1–H3**) in the network of **1** (viewing along the *b*-axis). (b) Electron density map *F*_0_ (blue mesh) contoured at the 1.0*σ* level overlaid on the structure of guest **H1**.

Thus, *ca.* 20 μg of a crude mixture obtained from the ether extract was purified using HPLC and subjected to guest soaking with a crystal of **1**. After guest soaking at 50 °C for 2 d, the crystallographic analysis revealed three independent molecules of **8** (**H1–H3** in Fig. 3a) in an asymmetric unit with occupancies of 100, 80.5, and 100%, respectively.¶
¶Crystallographic data for **1·8**: C_72_H_48_I_12_N_24_Zn_6_·2.8(C_19_H_26_O_3_)·1.5(C_6_H_12_), *M* = 4147.41, colorless plate, 0.28 × 0.08 × 0.07 mm^3^, monoclinic, space group *C*2, *a* = 34.7707(6) Å, *b* = 14.8406(3) Å, *c* = 31.2152(5) Å, *β* = 102.601(2)°, *V* = 15 719.6(5) Å^3^, *Z* = 4, *D*_c_ = 1.749 g cm^–3^, *T* = 93(2) K, 2.9030° < *θ* < 73.3970°, 29 574 unique reflections out of 106 306 with *I* > 2*σ*(*I*), GoF = 1.005, final *R* factors *R*_1_ = 0.0788, and w*R*_2_ = 0.2259 for all data, Flack parameter (Parsons) = –0.008(8), CCDC deposit number 1451763. All the structures revealed that the carbonyl group at C17 in **7** is stereoselectively reduced to a hydroxy group with an *S* configuration. The two carbonyl groups at C3 and C11 remain intact: typical C=O distances (1.19(3) and 1.20(3)) were observed at C3 and C11, respectively, and these carbon atoms still adopt a trigonal planar geometry. In contrast, elongation of the C–O bond length of 1.41(3) Å and a tetrahedral geometry at C17 were observed, indicating that the carbonyl reduction took place only at C17. Notably, favorable host–guest interactions were observed. For example, guest **H1** is trapped by the host framework of **1** with C=O···H–C or C–H···I hydrogen bond interactions.

## Conclusions

In conclusion, LC-SCD analysis has been demonstrated as a useful tool for structural analysis of trace microbial metabolites. Some important lessons are learned in this study working with trace microbial metabolites, which we have not noticed in the previous analysis of abundant synthetic compounds. First, a key to the success of the analysis is to obtain high purity samples of the microbial metabolites by HPLC separation. As the crystalline sponge **1** often preferentially absorbs minor components, even low-level impurities may disturb efficient guest soaking. Given that trace metabolites separated using HPLC are normally contaminated with many impurities, great care should be taken in the purification steps. Therefore, pre-purification with PTLC or LC–LC is highly recommended. Second, pre-analysis of the abundant parent compounds would be beneficial because soaking conditions optimized for the parent compounds can usually be applied to the analysis of their metabolites. Third, having successful results in this study, we are more convinced that the crystalline sponge method, coupled with HPLC separation, will innovate the structural analysis of scarce amounts of microbial products in natural product chemistry as well as in drug discovery.

## Supplementary Material

Supplementary information

Crystal structure data
