# Supplementary material for: Structure determination of microbial metabolites by the crystalline sponge method
Source: Chem Sci. 2016 Feb 24;7(6):3910–3. doi: 10.1039/c6sc00594b (PMC6013795; doi:10.1039/c6sc00594b)
Supplement: Supplementary file 1 [file SC-007-C6SC00594B-s001.pdf]

Electronic Supplementary Material (ESI) for Chemical Science.
This journal is © The Royal Society of Chemistry 2016

Supporting Information

Structure Determination of Microbial Metabolites by the Crystalline Sponge Method

Yasuhide Inokuma[1,2], Tomoya Ukegawa[1], Manabu Hoshino[1,3] and Makoto Fujita[1,3]*

[1]*Department of Applied Chemistry, School of Engineering, The University of Tokyo, 7-3-1 Hongo, Bunkyo-ku, Tokyo, 113-8656, Japan*
[2]*JST PRESTO, 4-1-8 Honcho, Kawaguchi, Saitama, 332-0012, Japan*
[3]*JST ACCEL, 4-1-8 Honcho, Kawaguchi, Saitama, 332-0012, Japan*

Table of Contents

I. **General Information**
II. **Analysis of Metabolites from Baker's Yeast by the Crystalline Sponge Method**
III. **References**

I. General Information

1. Reagents and equipment

Solvents and reagents were purchased from TCI Co., Ltd., WAKO Pure Chemical Industries Ltd., and Sigma-Aldrich Co and used without further purification. Dry yeast was purchased from Oriental Yeast Co., Ltd. Preparative thin layer chromatography (PTLC) was performed on a Merck Silica Gel 60 F_{254} Plate.

Analytical HPLC (high performance liquid chromatography) chromatograms were recorded on a JASCO MD-2018 photodiode array detecter equipped with a JASCO PU-2089 pump, JASCO AS-2059 sampler, JASCO CO-2060 column thermostat and ADVANTEC CHF 122SC fraction collector. For resolution of the metabolites, Inertsil NH2, Inertsil diol (inner diameter 4.6 mm × length 250 mm; GL Sciences), or Chiral Pak IC (inner diameter 4.6 mm × length 250 mm; Daicel) was used as a stationary phase. Microscopic IR spectra were recorded on a Varian DIGILAB Scimitar instrument and are reported in frequency of absorption (cm^{-1}). For single crystal X-ray diffraction analysis and microscopic IR measurement, fluorolube® and mineral oil were used as a protectant for the single crystals.

2. Single Crystal X-ray Analysis

Single crystal X-ray diffraction data were collected on a BRUKER APEX-II CCD diffractometer equipped with a focusing mirror (MoK_α radiation λ = 0.71073 Å) and a N_2 generator (Japan Thermal Eng. Co., Ltd.), Rigaku XtaLAB P200 diffractometer equipped with a PILATUS200K detector using multi-layer mirror (CuK_α radiation λ= 1.5418 Å) or Rigaku SuperNova equipped with a fine-focused X-ray source (CuK_α radiation λ= 1.5418 Å). All structures were solved using a dual-space algorithm (SHELXT[1]) and refined using full-matrix least-squares method (SHELXL[2]). All the non-hydrogen atoms for host framework were refined anisotropically.

3. Experimental settings for LC-SCD analysis

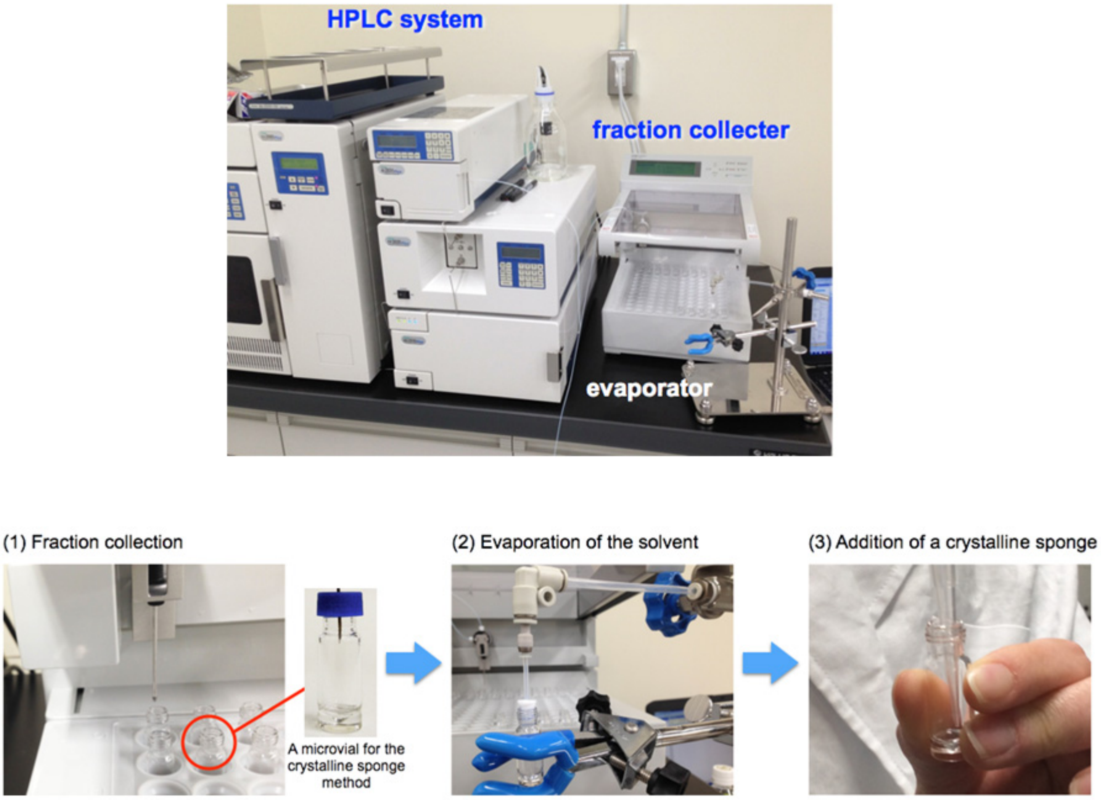

Figure S1. Experimental settings (top) and procedures (bottom) for the liquid chromatography–single crystal diffraction (LC-SCD) analysis of trace metabolites. (1) Fractions separated by analytical HPLC are directly collected in Screw-top microvials (Osaka chemical, cat. no. 11090620) . (2) Solvents are evaporated using an evaporator (BioChromato, Conveni Evapo C1). (3) To the residue, a crystalline sponge is added to carry out the crystalline sponge method.

II. Analysis of Metabolites from Baker's Yeast by the Crystalline Sponge Method

1. General procedure for the metabolism of organic substrates by baker's yeast

To a solution of sucrose (23.5 g, 68.7 mmol) in distilled water (100 ml), dry yeast (11.2 g) was added at 30 ºC and the resulting solution was stirred for 30 min. Substrate (100 mg) was then added to the solution and the resulting mixture was incubated at 30 ºC with stirring. For details of incubation time and workup procedure, see the following sections.

2. Metabolite of DDT

2-1. Metabolism of the substrate

According to the general procedure, DDT (**2**) was metabolized at 30 ºC for 7 d. From the resulting mixture, a 0.15 mL aliquot was sampled and crude metabolites were extracted with diethyl ether (1 ml × 3 times). The extract was then dried over Na_2SO_4 and the solvent was evaporated to leave oily residue. Here, the yield of the main metabolite **3** was determined to be 1.3% by the calibration curve using a standard compound purchased from TCI.

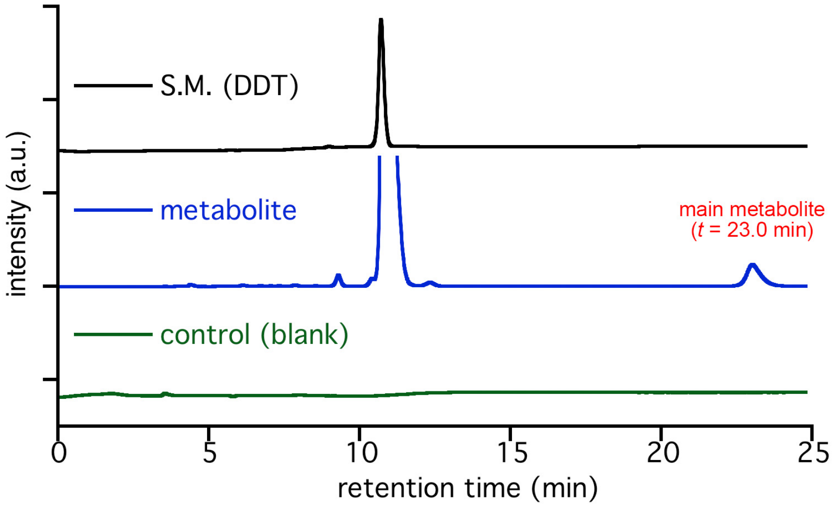

Figure S2. HPLC chromatograms of (black) the parent substrate DDT (**2**), (blue) crude metabolite, and (green) crude extract from blank control experiment (in he absence of DDT). (HPLC conditions; column: Inertsil diol, eluent: hexane, flow rate: 1 mL/min, detection: UV at 230 nm)

2-2. Sample preparation for LC-SCD Analysis

(1) Purification Method (i) (*successful example*)

The crude metabolites were pre-purified on PTLC using hexane as an eluent to remove impurities absorbed at the origin of the spot. The other fractions were extracted from silica gel plate with dichloromethane. After evaporation of the solvent, resulting residue was subjected to HPLC separation, and *ca*. 15 µg of metabolite **3** (t = 23.0 min in Fig. S2) was collected into a microvial for the crystalline sponge method. After solvent evaporation, a crystal of crystalline sponge **1** (0.23 × 0.11 × 0.09 mm^3) was added with 20 µl of cyclohexane. The microvial was sealed with a screw cap and incubated at r.t. for 1 d. The cap was then pierced with a syringe needle and the solvent was slowly and almost completely evaporated over 2 d at r.t. The resulting crystal was subjected to single crystal X-ray analysis.

(2) Purification Method (ii) (*unsuccessful example*)

From the crude mixture, *ca*. 15 µg of the main metabolite (t = 23.0 min) was collected in a microvial after 3 times separation by HPLC (400 µg of the mixture was injected in one batch; the metabolite was quantified by UV absorption at 254 nm using the calibration curve). The solvent was completely evaporated under the reduced pressure to leave an oily residue. Using the residue, crystalline sponge analysis was carried out under the same conditions above.

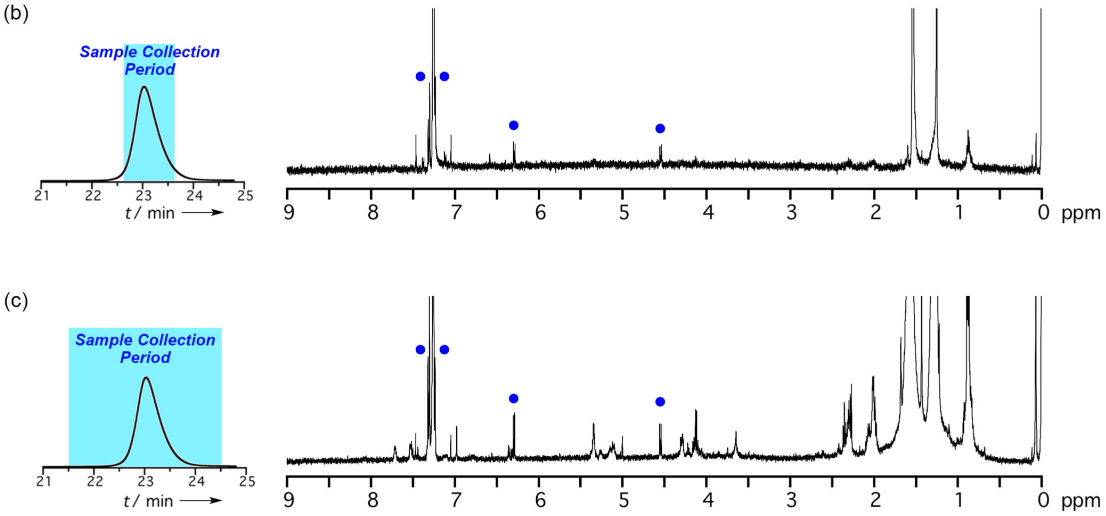

Figure S3. (left) The sample collection period and (right) ^{1}H NMR spectra of metabolite **3** obtained by HPLC separation using (a) purification method (i) and (b) purification method (ii) noted above. The NMR samples were prepared by repeated collection (~10 times) with the same HPLC conditions. Blue circles show the signals from metabolite **3**.

2-3. Crystallographic data

Crystallographic data for 1•3 prepared by method (i):

$C_{36}H_{24}N_{12}Zn_3I_6$•($C_{14}H_{10}Cl_4$)•($C_7H_4Cl_2$)•2.5($C_3H_6$), M = 2230.02, colorless block, 0.23 × 0.11 × 0.09 mm^3, Monoclinic, space group $C2/c$, a = 35.7365(13) Å, b = 15.0610(5) Å, c = 30.9345(11) Å, β = 104.141(7)°, V = 16145.2(11) Å^3, Z = 8, D_c = 1.835 g/cm^3, T = 93(2) K, 2.989° < θ < 27.468°, 17652 unique reflections out of 88524 with $I > 2\sigma(I)$, GoF = 1.063, final R factors R_1 = 0.0780, and wR_2= 0.2736 for all data, CCDC deposit number 1451761.

***Reference data*: Crystallographic data for 1•3 prepared by method (ii):**

$C_{36}H_{24}N_{12}Zn_3I_6$, M = 1528.18, colorless block, 0.23 × 0.11 × 0.09 mm^3, Monoclinic, space group $C2/c$, a = 34.700(7) Å, b = 14.749(3) Å, c = 31.478(6) Å, β = 101.964(3)°, V = 15760(5) Å^3, Z = 8, D_c = 1.334 g/cm^3, F000 = 7536, μ = 3.378 mm^{-1}, T = 93(2) K, 1.200° < θ < 27.551°, 17978 unique reflections out of 89896 with $I > 2\sigma(I)$, GoF = 1.050, final R factors R_1 = 0.0897, and wR_2= 0.3329 for all data. Because of the weak residual electron densities in the F_o–F_c map (the most intense peak: 1.67 e/Å^3, 2022 electron count/cell for solvent accessible void 7821.3 Å^3), guest molecules in the interstitial pores could not be modeled.

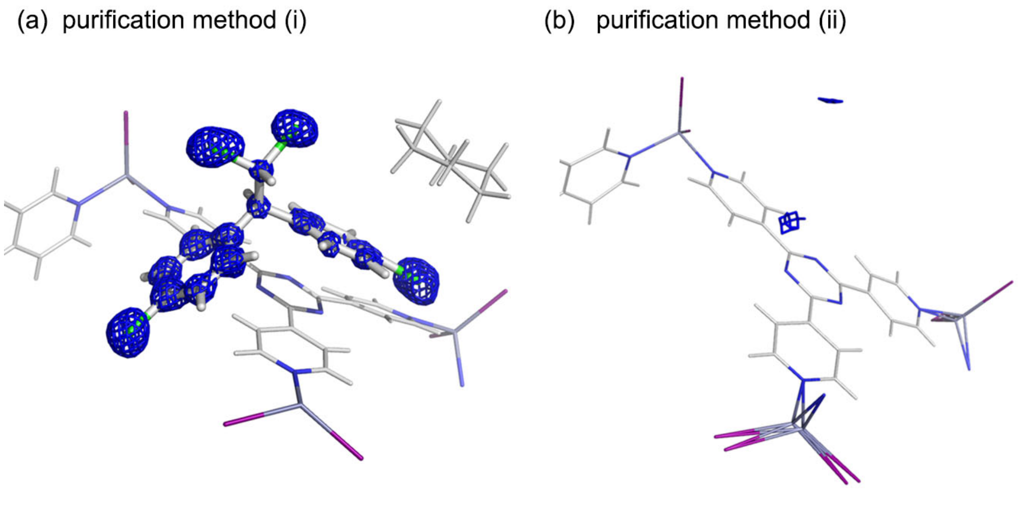

Figure S4. (a) Electron density maps F_o around the guest **3** binding site in crystalline sponge **1** for inclusion crystals **1•3** prepared by (a) method (i) and (b) method (ii) contoured at 0.8 and 0.3σ level, respectively.

Refinement details for DDD-included crystalline sponges

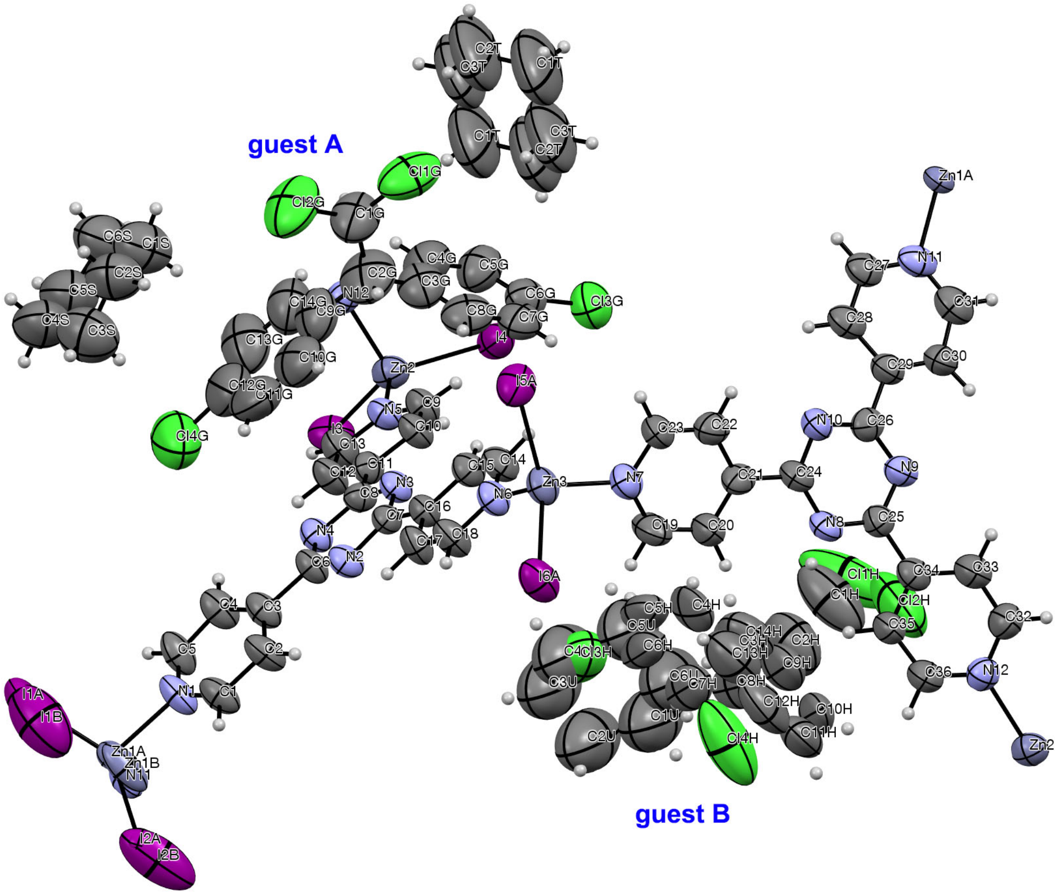

Figure S5. The asymmetric unit for DDD-included crystalline sponge **1•3** (prepared by method (i)). ORTEP drawing are set at the 50% probability.

In the asymmetric unit, two DDD molecules (labeled 'G' and 'H') were observed. The aromatic rings for DDD guests were constrained by AFIX 66 to generate ideal benzene rings. Since thermal motion of the weakly bound guests resulted in large ADPs, thermal factors of DDD guest G was partially (for C9G > C14G) restrained using DELU with a normal standard deviation. Guest H was disordered close to the twofold axes. We made a disordered model for guest H as shown in Fig. S6 with 50% occupancy each, and the structure was refined with minimal restraints DFIX and SIMU.

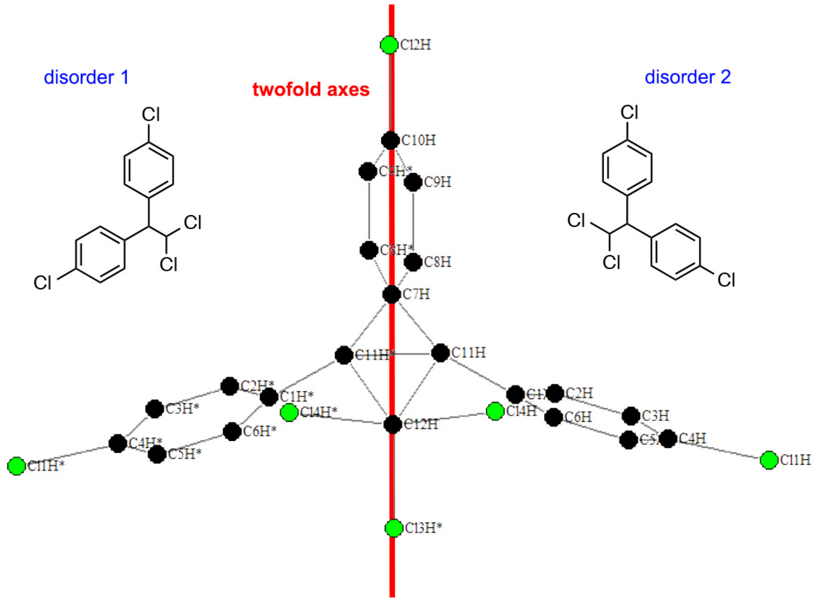

Figure S6. Disordered model used for the refinement of guest H.

Most of restraints were applied for cyclohexane solvents (labeled ‘S’, ‘T’, and ‘U’) that are highly disordered in the pores. Because of their flexible and weakly binding nature, geometrical restraints (DFIX, SADI and SAME) and SIMU were used for the least-square refinement.

3. Metabolite of tetralone 5

3-1. Metabolism of the substrate

According to the general procedure, methyl 5-methoxy-2-tetralone-1-carboxylate (**5**) (100 mg; added as an ethanol (2.5 mL) solution) was metabolized using 11.2 g of dry yeast and 23.5 g of D-sucrose at 30 ºC. After 18 h incubation, 100 mL of diethyl ether was added to the solution, and the resulting mixture was stirred overnight. Metabolites were then extracted with diethyl ether (100 mL × 3 times), washed with brine, dried over Na_2SO_4, and the solvent was evaporated to leave crude metabolites (*ca.* 450 mg) as an oily residue.

3-2. LC-SCD Analysis

The residue obtained above (*ca.* 10 mg) was dissolved in 1 mL of dichloromethane. After the filtration through a membrane filter, 20 µL of the sample solution was subjected to HPLC separation. Approximately 10 µg of the main metabolite (t = 15.2 min; HRMS: m/z = 259.0932, calculated for $C_{13}H_{16}NaO_4^+$: 259.0946) was collected in a microvial by HPLC. To the microvial, crystalline sponge **1** (480 × 70 × 50 µm^3), cyclohexane (45 µL) and 1,2-dichloroethane (5 µL) were added. The microvial was then sealed with a screw cap and incubated at 50 ºC for 4 d. The cap was then pierced with a syringe needle and the solvent was slowly and almost completely evaporated over 2 d at 50 ºC. The resulting crystal was subjected to single crystal X-ray analysis. Note that the metabolite obtained under these conditions was enough pure to be used for the crystalline sponge analysis without further purification.

Metabolite **6**: ^{1}H NMR (500 MHz, $CDCl_3$, 300 K): δ 7.14 (t, J = 8.0 Hz, 1H, Ar-H), 6.79 (d, J = 8.0 Hz, 1H, Ar-H), 6.74 (d, J = 8.0 Hz, 1H, Ar-H), 4.20 (m, 1H, C*H*-OH), 4.02 (d, J = 5.0 Hz, 1H, C*H*-CO_2Me), 3.82 (s, 3H, OMe), 3.76 (s, 3H, OMe), 2.97 (dt, J = 5.5 and 18.5 Hz, benzyl-CH_2), 2.91 (br. d, 1H, OH), 2.66 (dt, J = 8 and 17.5 Hz, benzyl-CH_2), 2.23 (m, 1H, methylene), 2.23 (m, 1H, methylene), and 1.99 (m, 1H, methylene).

3-3. Synthesis of reference compounds by $NaBH_4$ reduction

To confirm the optical resolution on chiral stationary phase, we synthesized reference compounds, namely, a racemic mixture of *cis*- and *trans*-methyl 2-hydroxy-5-methoxy-1,2,3,4-tetrahydronaphthalene-1-carboxylate. To a solution of tetralone **5** (15 mg, 64 µmol) in methanol (1 mL), $NaBH_4$ (4.9 mg) was added. After stirring for 40 min at r.t., the reaction was quenched with sat. NH_4Cl aq. (5 mL). Products were extracted with CH_2Cl_2. The organic extract was

washed with brine, dried over Na_2SO_4, and the solvent was evaporated. The crude reaction mixture was chromatographed on a TLC plate (20 × 20 cm; eluent: hexane/ethyl acetate = 1:1) to give the target compounds as a mixture of diastereomers (9.9 mg; *cis*/*trans* = 93:7, determined by NMR and HPLC analysis).

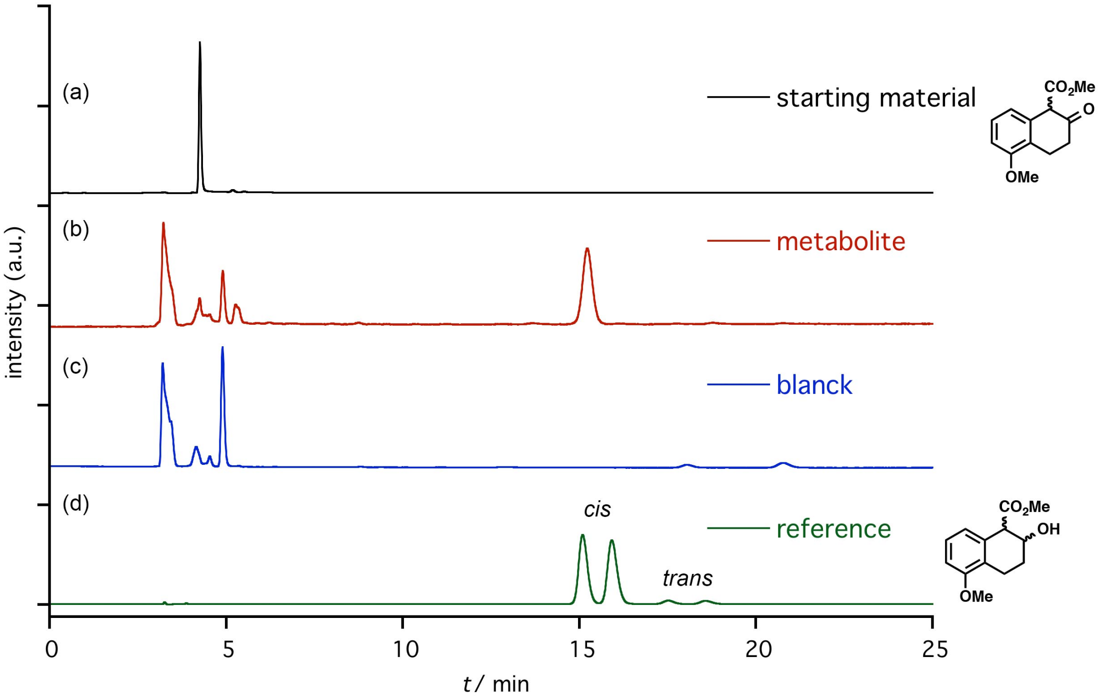

Figure S7. HPLC chromatograms of (a) tetralone **5**, (b) crude metabolite, (c) crude extract from control experiment (in the absence of tetralone **5**), and (d) synthetic mixture of the corresponding alcohol obtained by $NaBH_4$ reduction as a reference. (HPLC conditions; column: Chiralpak IC, hexane/CH_2Cl_2/EtOH = 94:1:5, flow rate: 1 mL/min, detection: UV at 270 nm)

3-4. Crystallographic data

Crystallographic data for 1•6: $C_{72}H_{48}I_{12}N_{24}Zn_6$•3.79($C_{13}H_{16}O_4$)•1.93($C_6H_{12}$), M = 4220.24, colorless rod, 0.18 × 0.11 × 0.06 mm^3, Monoclinic, space group $C2$, a = 36.3783(8) Å, b = 14.6755(2) Å, c = 31.2278(6) Å, β = 103.184(2)°, V = 16232.2(5) Å^3, Z = 4, D_c = 1.727 g/cm^3, T = 100(2) K, 4.1720° < θ < 74.0140°, 31961 unique reflections out of 69980 with $I > 2\sigma(I)$, GoF = 1.031, final R factors R_1 = 0.0511, and wR_2= 0.1396 for all data, Flack parameter (Parsons) = 0.010(4), CCDC deposit number 1451762.

Microscopic IR (single crystal, mineral oil): 2927 (br), 2852 (m), 1733 (m), 1670 (m), 1618 (m) 1586 (m), 1507(m), 1421 (m) and 1372 (m) cm^{-1}.

Refinement details for compound 6-included crystalline sponges

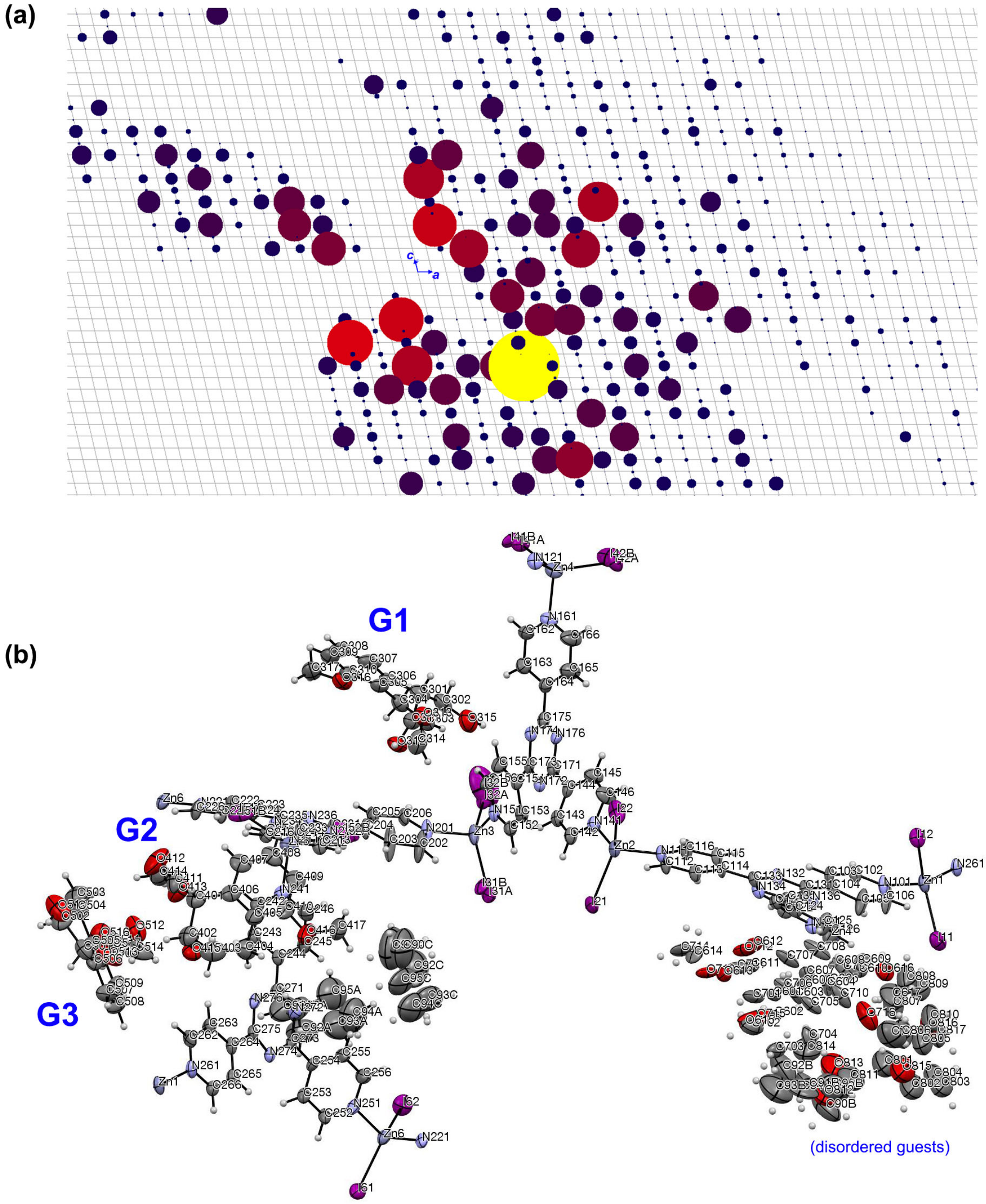

Figure S8. (a) Reciprocal space of inclusion crystal **1•6** (Bragg spots in the *h0l* plane) (b) The asymmetric unit for compound **6**-included crystalline sponge **1•6**. ORTEP drawings are set at the 50% probability.

The absence of *c*-glide plane was indicated by clear appearance of $h0l$ (l = odd number) reflections (Fig S8(a)). Therefore, data reduction was performed using non-centrosymmetric space group *C*2.

In the pore space, three guest molecules **6** were observed (G1: C301~C317, G2: C401~C417, and G3: C501~C517) and their structures were refined with 100% occupancy without any geometrical restraints. Another guest molecule of **6** seems to exist in the remaining void with low occupancy (~25%), but this guest was not properly refined due to severe disorder with a cyclohexane solvent. Note that extraction experiment using inclusion crystals **1•6** confirmed that there is no possibility of contamination of other diastereomer/enantiomer or other impurities.

4. Metabolite of adrenosterone (7)

4-1. Metabolism of the substrate

Adrenosterone (**7**) (0.200 mg; added as an ethanol (0.1 mL) solution) was metabolized using 61.0 mg of dry yeast and 134 mg of D-sucrose in 1.0 mL of distilled water at 30 ºC. After 4 h incubation, 1.0 mL of diethyl ether was added to the solution, and the resulting mixture was stirred overnight at r.t. Metabolites were then extracted with diethyl ether (1.0 mL × 3 times), washed with brine, dried over Na_2SO_4, and the solvent was evaporated to leave crude metabolites (*ca.* 2.9 mg) as an oily residue.

4-2. LC-SCD Analysis

To the entire residue obtained above, was added 0.2 mL of acetonitrile, and the insoluble materials were removed by filtration through a membrane filter. An approximately 20 μL aliquot was injected to HPLC and main metabolite **8** (*t* = 21.7 min; HRMS: *m/z* = 325.1781, calculated for $C_{19}H_{26}NaO_3^+$: 325.1780)) was collected in a microvial by HPLC. To the microvial, crystalline sponge **1** (110 × 110 × 50 μm^3), cyclohexane (45 μL) and 1,2-dichloroethane (5 μL) were added. The microvial was then sealed with a screw cap and incubated at 50 ºC for 5 d. The cap was then pierced with a syringe needle and the solvent was slowly and almost completely evaporated over 2 d at 50 ºC. The resulting crystal was subjected to single crystal X-ray analysis. Note that the metabolite obtained under these conditions was enough pure to be used for the crystalline sponge analysis without further purification.

Metabolite **8**: ^1H NMR (500 MHz, $CDCl_3$, 300 K): δ 5.72 (s, 1H, vinylic), 3.87 (br, 1H, C*H*-OH), 2.77 (m, 1H), 2.51–2.38 (m, 3H), 2.32–2.28 (m, 2H), 2.22–2.17 (m and d, 2H), 2.00–1.92 (m, 2H), 1.88 (d, *J* = 11 Hz, 1H), 1.78 (m, 1H), 1.68–1.52 (m, 3H), 1.45–1.37 (m and s, 5H), 1.28–1.18 (m, 1H), and 0.76 (s, 3H, Me).

The ^1H NMR signals matched with the reported data.[3]

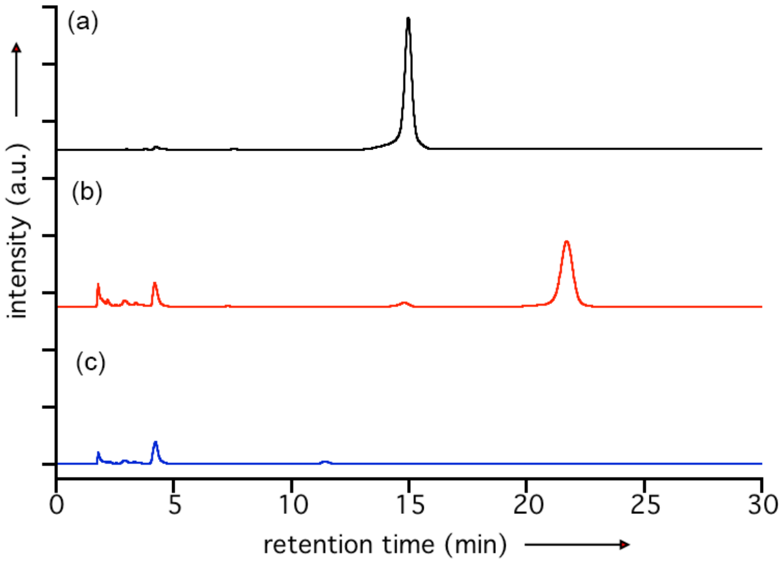

Figure S9. HPLC chromatograms of (a) adrenosterone (**7**), (b) crude metabolite, and (c) crude extract from control experiment (in the absence of compound **7**). (HPLC conditions; column: Chiralpak IC, hexane/EtOH = 14:1, flow rate: 1 mL/min, detection: UV at 238 nm)

Crystallographic data for 1•8: $C_{72}H_{48}I_{12}N_{24}Zn_6$•2.8($C_{19}H_{26}O_3$)•1.5($C_6H_{12}$), M = 4147.41, colorless plate, 0.28 × 0.08 × 0.07 mm^3, Monoclinic, space group $C2$, a = 34.7707(6) Å, b = 14.8406(3) Å, c = 31.2152(5) Å, β = 102.601(2)°, V = 15719.6(5) Å^3, Z = 4, D_c = 1.749 g/cm^3, T = 93(2) K, 2.9030° < θ < 73.3970°, 29574 unique reflections out of 106306 with $I > 2\sigma(I)$, GoF = 1.005, final R factors R_1 = 0.0788, and wR_2= 0.2259 for all data, Flack parameter (Parsons) = –0.008(8), CCDC deposit number 1451763.

Microscopic IR (single crystal, fluorolube): 2922 (m), 2850 (m), 1775 (m), 1732 (m), 1701 (m), 1619 (m), 1576 (m), 1515 (m), 1452 (m) and 1378 (m) cm^{-1}.

Refinement details for metabolite 8-included crystalline sponge

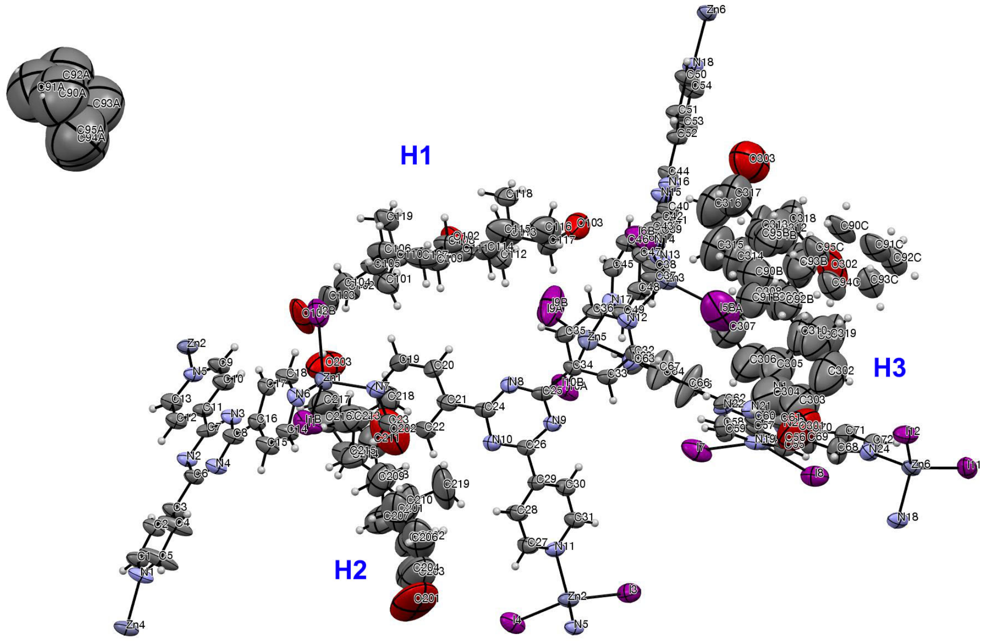

Figure S10. The asymmetric unit for metabolite **8**-included crystalline sponge **1•8**. ORTEP drawings are set at the 50% probability.

The structural analysis was preformed in the non-centrosymmetric space group *C*2. All the non-hydrogen atoms were refined anosotropically. In the void space of the asymmetric unit, three guests of **8** were observed (H1: C101~O103, H2: C201~O203, and H3: C301~O303). Guest H1 and H3 were refined with 100% occupancy, while the occupancy of H2 was estimated to be 80.5% by free refinement using a unique site occupancy factor as performed in the previous report by Ramadhar *et al.*[4] As the purity of guest compound **7** was confirmed by NMR and HPLC analyses, guests **H2** and **H3** were geometrically restrained by SAME command with a standard deviation of 0.02. Due to severe disorder and low bond precision, short inter/intra H•••H contact alerts arise for the cyclohexane solvent.

5. NMR analysis of the metabolites (reference experiment)

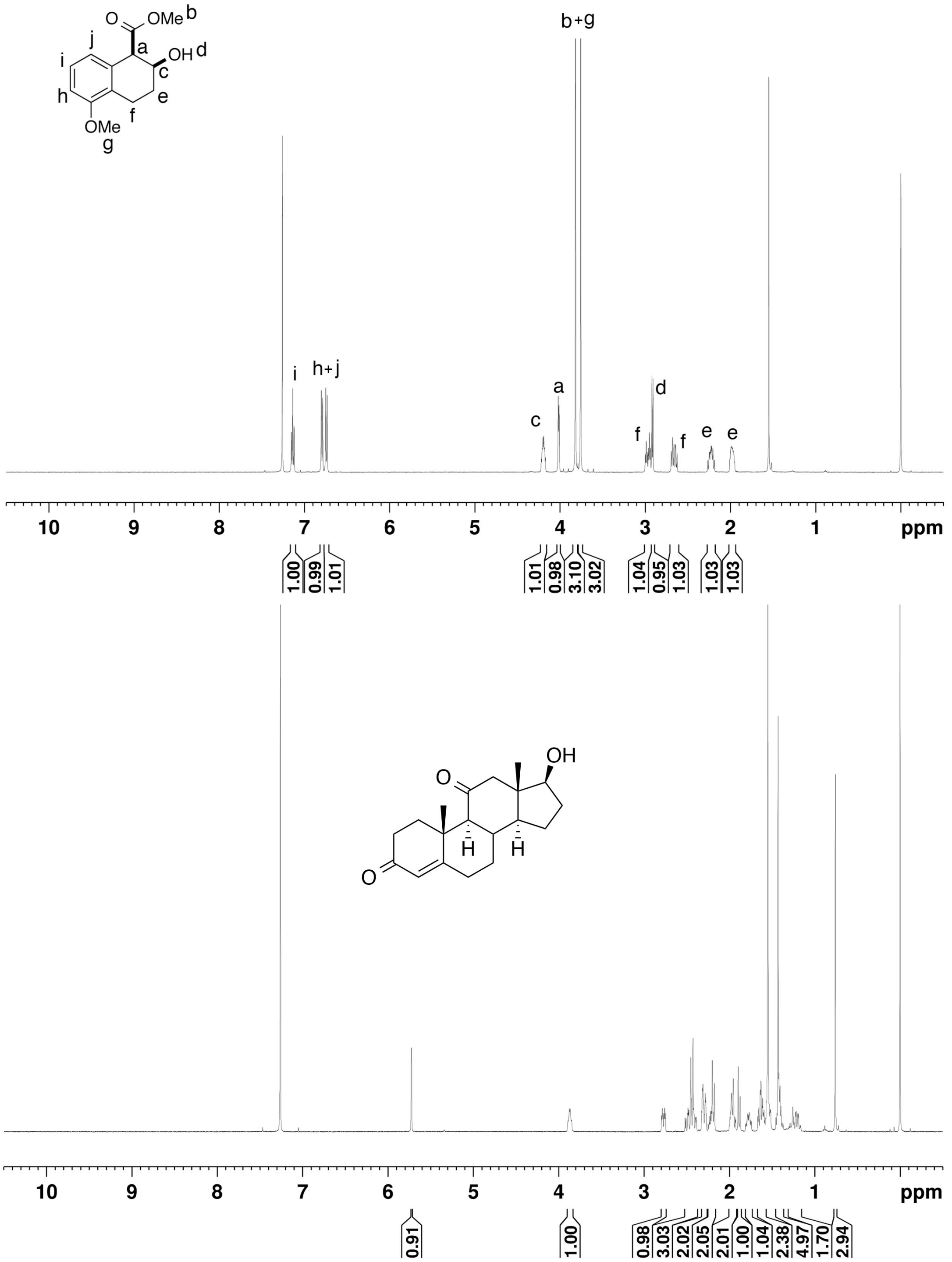

Figure S11. ^{1}H NMR spectra (500 MHz, $CDCl_3$) of metabolites **5** and **7**. For the NMR analysis, samples of the metabolites were separated on a milligram scale.

References

1. G. M. Sheldrick, *Acta Crystallogr. Sect. A* **2015**, 71, 3-8.
2. G. M. Sheldrick, *Acta Crystallogr. Sect. C* **2015**, 71, 3-8.
3. A. C. Hunter, J. Elsom, L. Ross, R. Barrett *Biochimica et Biophysica Acta*, **2006**, *1761*, 360–366.
4. T. R. Ramadhar, S.-L. Zheng, Y.-S. Chen, J. Clardy *Acta Cryst.* **2015**, *A71*, 46-58.
